# Type 2 Marine-Lenhart Syndrome: An Uncommon Cause of Thyrotoxicosis

**DOI:** 10.7759/cureus.20558

**Published:** 2021-12-21

**Authors:** Carlos Alfonso Builes Barrera, Pablo Alberto Castaño, Paula Herrera Revollo, Marcel Eduardo Pérez Paternina, Luis Antonio Rodriguez Arrieta

**Affiliations:** 1 Internal Medicine and Endocrinology, University of Antioquia, San Vicente Fundación Hospital, Medellín, COL; 2 Endocrinology, Military University of Nueva Granada, Medellín, COL; 3 Endocrinology and Metabolism, University of Antioquia, Medellín, COL; 4 General Medicine, Libre University of Barranquilla, Barranquilla, COL; 5 General Medicine, University of Magdalena, Santa Marta, COL

**Keywords:** radioactive iodine, diffuse goiter, hyperthyroidism, thyrotoxicosis, marine-lenhart syndrome

## Abstract

Marine-Lenhart syndrome (MLS) is an uncommon cause of primary hyperthyroidism, which can occur in the context of diffuse goiter due to Graves disease (GD) or autonomic nodular disease (Plummer disease (PD)). The coexistence of these two conditions is the hallmark of the MLS. Patients with MLS have a lower remission rate with oral antithyroid drugs, requiring definitive management therapies with radioactive iodine or surgery. We present the case of a 48-year-old female with a history of primary autoimmune hyperthyroidism (GD) since 2016, with biochemical control of hyperthyroidism with methimazole but without the possibility of stopping treatment. The scintigraphic uptake pattern showed heterogeneous uptake of the thyroid parenchyma with three hyper-uptake nodules without inhibition of the rest of the thyroid tissue, findings of an MLS condition with the indication for definitive therapy, for which he was referred to nuclear medicine for the administration of radioactive iodine.

## Introduction

Thyrotoxicosis is the set of symptoms and signs associated with an excess circulation of thyroid hormones, whether of thyroid or extrathyroidal origin [1]. Primary hyperthyroidism is endogenous thyrotoxicosis due to excess activity of the thyroid, with hyperthyroidism of autoimmune origin being the main cause of non-nodular hyperthyroidism, which when presenting with diffuse hyper-uptake goiter and exophthalmos represents the hallmark of Graves disease (GD) [1,2]. In turn, Plummer disease (PD) is an entity characterized by the presence of autonomous thyroid nodules, also called “hot autonomous nodules or toxic adenomas (TA)” (due to their hyper-uptake of the radiotracer observed in thyroid scintigraphy), causing primary hyperthyroidism mainly in the population older than 50-60 years, in contrast to the higher prevalence of GD between 20 and 30 years [2,3]. However, it has been determined that the coexistence of nodules can occur in up to 35% of patients with GD, and 0.8%-2.7% of cases are functional adenomas; this combination has been called Marine-Lenhart syndrome (MLS) [4,5]. This syndrome is rare and occurs in 1%-2.7% of all patients with GD [6]. The absence of ocular involvement in a patient with diffuse goiter in addition to nodules on ultrasound and a scintigraphic pattern with hot nodules accompanied by hyper-uptake thyroid tissue can help identify this syndrome, with prognostic relevance due to its lower remission rate, with the use of antithyroid drugs and the need for higher doses of radioiodine than in other hyperthyroidism scenarios [6,7]. We describe the case of a 48-year-old female with primary hyperthyroidism initially approached as a GD, with scintigraphic findings suggestive of type 2 MLS.

## Case presentation

This case report is of a 48-year-old female with primary hyperthyroidism initially with TSH of 0.001 mUI/L (reference value: 0.5-4.0 mUI/L), free thyroxine (FT4) of 3.4 ng/dL (reference value: 0.7-2.0 ng/dL), and diffuse goiter, without clinical thyroid orbitopathy, diagnosed since 2016. She started endocrinology follow-up and received treatment with methimazole between 5 and 15 mg/day and propranolol 40 mg/day. However, after receiving treatment for more than 48 months with adequate adherence, treatment could not be successfully withdrawn, and reactivation of hyperthyroidism was avoided by reducing the antithyroid dose.

Thyroid ultrasound reported diffuse goiter and the presence of three bilateral, solid, isoechoic thyroid nodules, with the increased central flow on color Doppler, without calcifications, with regular borders; two nodules were located in the middle portion and in the lower pole of the right thyroid lobe (TL) and another nodule in the middle of the left TL. The lower right major nodule measured 1.3 × 1.1 × 0.8 cm.

**Figure 1:**
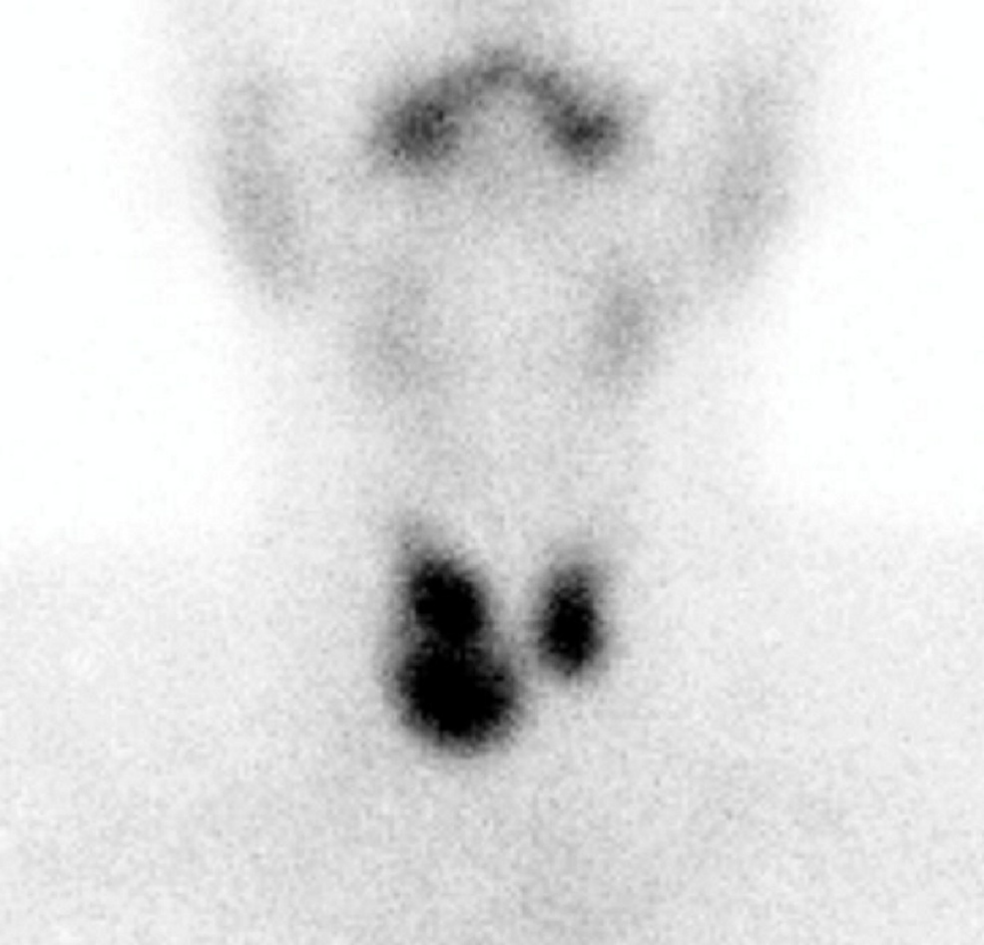
Thyroid scintigraphy Thyroid scintigraphy shows enlarged TL, with an increase in the Tc99m entrapment index; three hyper-uptake nodules are distinguished, two in the right TL and one in the left TL, without inhibiting the uptake of the rest of the gland tissue.

**Figure 2:**
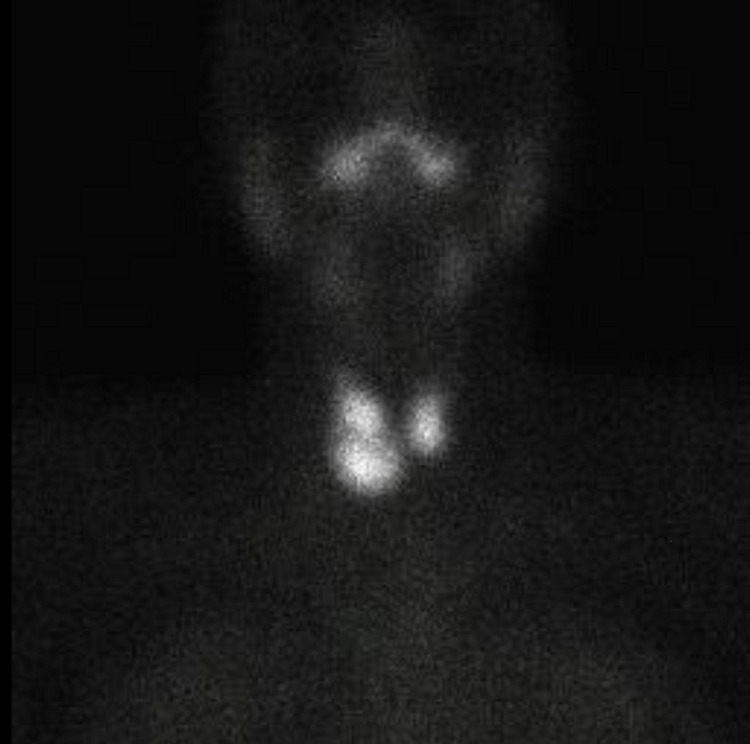
Thyroid scintigraphy Thyroid scintigraphy shows a pattern of uptake corresponding to type 2 MLS.

The absence of exophthalmos associated with goiter with thyroid nodules and the lack of control of the disease after more than 18 months of treatment led to requesting levels of anti-TSH receptor antibodies (TRAbs) that were not carried out due to administrative procedures. Additionally, thyroid scintigraphy with technetium 99 (Tc99m) was requested in December 2019 to define the uptake pattern. Due to the COVID-19 pandemic, the patient did not continue follow-up until October 2021. The report of the thyroid scintigraphy with Tc99m described that both TLs increased in size, with an increase in the entrapment index of 8% (normal value of 3.5%-5%). The parenchyma had a heterogeneous uptake with three hyper-uptake nodules, two in the right TL and one in the left TL without inhibiting the rest of the gland tissue (Figure 1 and Figure 2).

Based on this result, the diagnosis of type 2 MLS was made. Due to the use of methimazole for more than four years, the possibility of definitive therapy (radioactive iodine therapy or total thyroidectomy) for the management of her condition was raised. The patient was referred to nuclear medicine for management with iodotherapy.

## Discussion

Doctors David Marine and Carl H. Lenhart in Ohio, United States, 110 years ago, published a series of cases with exophthalmic goiter (corresponding to GD), finding hyper-uptake nodules in eight of them, which is why it was considered a rare and interesting variant of this disease [8,9]. In 1972, Charkes found 10 cases (2.7%) of functioning nodules in a series of 375 patients with GD and was the first to introduce the eponymous “Marine-Lenhart syndrome.” This condition is estimated to occur in 0.8%-2.7% of patients with GD [10].

Within the pathophysiological aspects, a unifying hypothesis has been proposed, which suggests that in patients with hyperfunctioning diffuse goiters, the preferential development of diffuse or nodular follicular hyperplasia may depend on the intrinsic function, concentration, and higher selective affinity of receptor-stimulating immunoglobulins of TSH that allow the development of TA [3-11].

**Figure 3:**
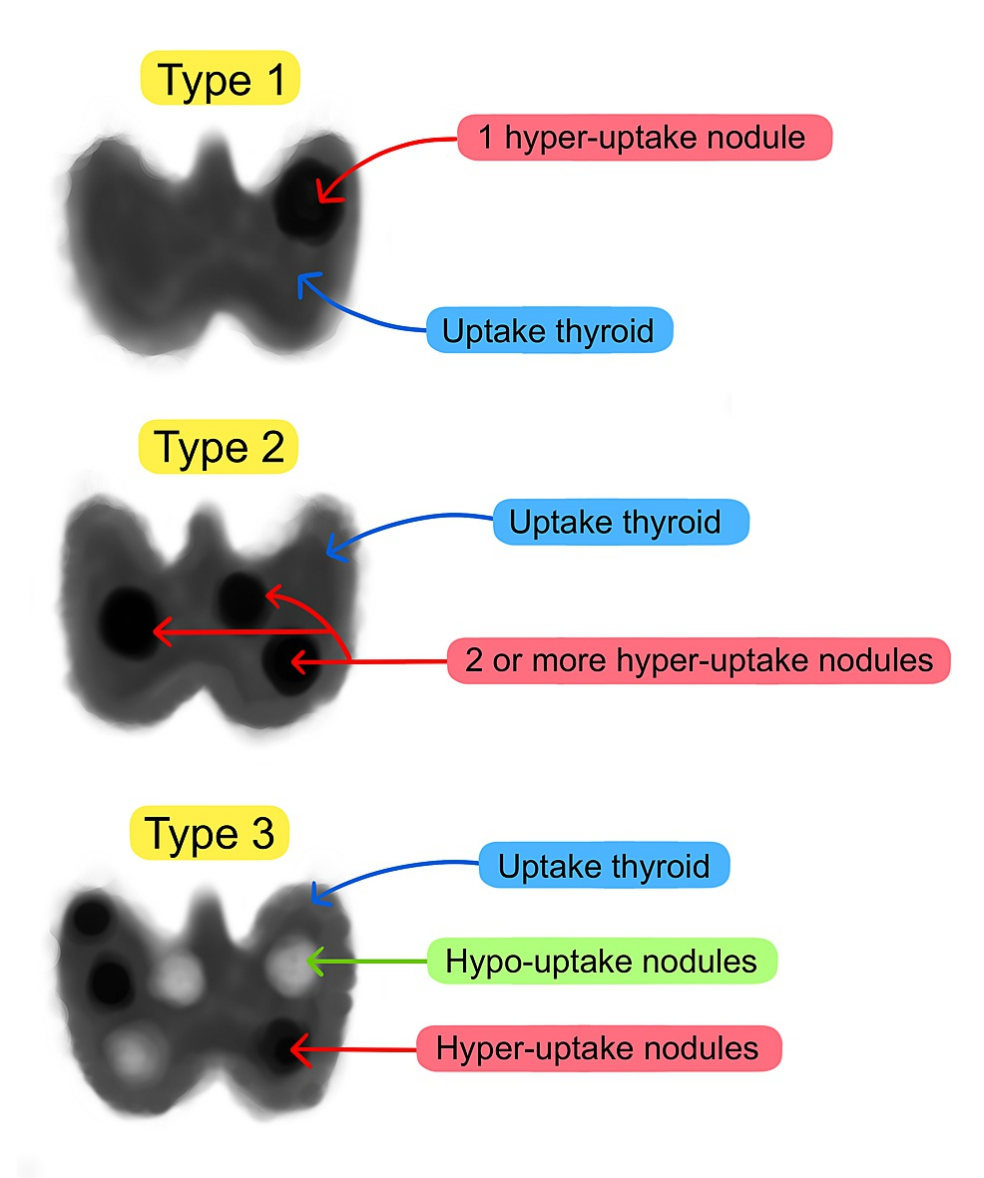
Classification of scintigraphic uptake patterns The scintigraphic pattern reported in the case corresponds to a type 2 pattern. Source: Authors Illustrated by Sustacia P.

Regarding the diagnosis, the possibility of identifying thyroid nodules in GD is 10%-15%; however, the challenge is to determine how many of these nodules show uptake by autonomy [1,2,11]. Thyroid scintigraphy is a study that allows the diagnosis of this syndrome, especially since we want to highlight that MLS has three main subtypes of scintigraphic pattern: type 1, high-uptake thyroid and single nodule of autonomous function; type 2, a pattern showing the thyroid with high uptake and several TA; and type 3, which presents the characteristics of the previous pattern adding up cold nodules (Figure 3). All these variants, as in the reported case, are accompanied by hyper-uptake thyroid parenchyma that is not inhibited in the presence of TA; for example, in our case, three hot nodules were evidenced [12] (Figure 3).

Regarding its treatment, the use of oral antithyroid drugs hardly allows a remission of the disease, and definitive therapy with radioactive iodine generally warrants higher doses when compared with other patients with hyperthyroidism of other etiologies [13,14]. Additionally, prolonged use of methimazole and propylthiouracil can cause rare serious adverse effects such as agranulocytosis, vasculitis, and liver dysfunction. As a consequence of the above, the hyperthyroidism management guidelines and the nuclear medicine guidelines consider patients with MLS as candidates for iodotherapy, in the absence of contraindications for its use [15,16].

In line with what has been discussed, it is important to elucidate the question: Is having a Marine-Lenhart syndrome the same as Graves disease? It is precisely this case that guides us to establish differences, and despite having similar clinical manifestations, it is important to specify that MLS is a condition that marks prognosis and that, unlike cases with GD that can achieve remission with the use of antithyroid drugs for 18-24 months, in patients with MLS, the option of definitive therapy should be motivated, such as how the patient of the case was proceeded [1-17,18].

One of the limitations identified in this case report is that anti-TSH receptor antibodies were requested; however, at the time of publishing this report, the results were not yet available to confirm the diagnosis of hyperthyroidism of autoimmune origin. Concerning these antibodies, if the patient had reported a positive value with high titers, the need to not continue medical therapy with antithyroid drugs and to consider the use of definitive therapy would be confirmed [1,2].

## Conclusions

The use of diagnostic tests in nonmalignant diffuse thyroid pathologies, both biochemical and imaging, allows the characterization and precision of patients with thyrotoxicosis pictures. Regardless of its low frequency, this syndrome should be considered among the options when approaching a patient with thyrotoxicosis. We report this case with a type 2 MLS and highlight the importance of this diagnosis in clinical decision-making to guide definitive treatment.
